# Efficacy, tolerability, and safety of erenumab for the preventive treatment of persistent post-traumatic headache attributed to mild traumatic brain injury: an open-label study

**DOI:** 10.1186/s10194-020-01136-z

**Published:** 2020-06-03

**Authors:** Håkan Ashina, Afrim Iljazi, Haidar Muhsen Al-Khazali, Anna Kristina Eigenbrodt, Eigil Lindekilde Larsen, Amalie Middelboe Andersen, Kevin John Hansen, Karoline Bendix Bräuner, Thomas Mørch-Jessen, Basit Chaudhry, Sonja Antic, Casper Emil Christensen, Messoud Ashina, Faisal Mohammad Amin, Henrik Winther Schytz

**Affiliations:** 1 Danish Headache Center, Department of Neurology, Rigshospitalet Glostrup, Faculty of Health and Medical Sciences, University of Copenhagen, Valdemar Hansens Vej 5, DK-2600 Glostrup, Denmark; 2 Department of Neurology, Aarhus University Hospital, Aarhus, Denmark

**Keywords:** Concussion, Secondary headache, Head trauma, Head injury, Clinical management

## Abstract

**Background:**

Calcitonin gene-related peptide (CGRP) has recently been implicated in the pathogenesis of post-traumatic headache (PTH), which raises the prospect for therapeutic use of monoclonal antibodies targeting CGRP or its receptor. Therefore, we decided to assess the efficacy, tolerability, and safety of erenumab for prevention of persistent PTH attributed to mild traumatic brain injury.

**Methods:**

A single-center, non-randomized, single-arm, open-label study of erenumab for adults aged 18–65 years with persistent PTH. Patients were assigned to receive 140-mg erenumab monthly by two subcutaneous 1-mL injections, given every 4 weeks for 12 weeks. The primary outcome measure was the mean change in number of monthly headache days of moderate to severe intensity from baseline (4-week pretreatment period) to week 9 through 12. Tolerability and safety endpoints were adverse events (i.e. number and type).

**Results:**

Eighty-nine of 100 patients completed the open-label trial. At baseline, the mean monthly number of headache days of moderate to severe intensity was 15.7. By week 9 through 12, the number was reduced by 2.8 days. The most common adverse events were constipation (n = 30) and injection-site reactions (n = 15). Of 100 patients who received at least one dose of erenumab, two patients discontinued the treatment regimen due to adverse events.

**Conclusions:**

Among patients with persistent PTH, erenumab resulted in a lower frequency of moderate to severe headache days in this 12-week open-label trial. In addition, erenumab was well-tolerated as discontinuations due to adverse events were low. Placebo-controlled randomized clinical trials are needed to adequately evaluate the efficacy and safety of erenumab in patients with persistent PTH.

**Trial registration:**

ClinicalTrials.Gov, NCT03974360. Registered on April 17, 2019 - Retrospectively registered

## Introduction

Post-traumatic headache (PTH) is a common sequela of mild traumatic brain injury (TBI) [1, 2], with a lifetime prevalence of 4.7% in men and 2.4% in women [3]. In addition, it has been documented that symptoms suggestive of depression, sleep disturbances, and post-traumatic stress disorder are frequent following mild TBI [2, 4, 5], including in those who develop persistent PTH [6, 7]. A nationwide registry-based study has also found an increased suicide risk in individuals with TBI, compared with the general population without TBI [8]. Despite the widespread prevalence and disability associated with PTH [4], there is little evidence to support any acute or preventive medication therapy [5]. In fact, no pharmacological agent has been approved for the treatment of PTH. As such, clinicians often choose a preventive treatment based on the individual patients’ headache phenotype. This approach has not been systematically investigated and, thus, lacks evidence. Taken together, there remains a considerable unmet need for mechanism-based treatments that are effective and well-tolerated. In this context, monoclonal antibodies targeting calcitonin gene-related peptide (CGRP) or its receptor might hold great promise as PTH often mimics a migraine-like headache [6] and anti-CGRP monoclonal antibodies have proven effective for preventive treatment of migraine [7–11]. In addition, preclinical data have emerged and demonstrated hypersensitivity to CGRP in concussed rodents [12, 13]. Thus, we find it timely to assess the efficacy, tolerability, and safety of erenumab for preventive treatment of persistent PTH attributed to mild TBI.

## Methods

### Study oversight

This trial was approved by the Regional Health Research Ethics Committee of the Capital Region of Denmark (identifier: H-18050498). In addition, study approval was also obtained from the Danish Medicines Agency (identifier: 2018–1104) and the Danish Data Protection Agency (identifier: VD-2019-20). Written informed consent was obtained from each participant before any study procedures or assessments were performed. Moreover, this trial was conducted in accordance with the Declaration of Helsinki [14].

### Study participants

Patients were recruited from the outpatient clinic of the Danish Headache Center and from neurological departments and rehabilitation centers in the Capital Region of Denmark as well as the Region of Southern Denmark. Patients included males and females aged 18 to 65 years with a history of persistent headache attributed to mild TBI in accordance with the 3rd edition of the International Classification of Headache Disorders (ICHD-3), [15]. Patients were allowed to use one concomitant preventive headache medication taken at a stable dose, i.e. no changes to the dose within 2 months before the baseline phase. In addition, patients were required to maintain stable dosing during the baseline phase and throughout the treatment phase. Use of acute headache medications was permitted, although patients with medication-overuse headache were excluded. Patients were also excluded if they had any history of primary headache disorder, except infrequent tension-type headache (TTH), or any history of whiplash injury. The complete list of inclusion and exclusion criteria is available in Supplement 1.

### Study design and procedures

This non-randomized, single-arm clinical trial consisted of a screening phase (0 to 2 weeks), baseline phase (4 weeks), and an open-label treatment phase (12 weeks). The patients had five scheduled study site visits: screening, baseline (dose 1), week 4 (dose 2), week 8 (dose 3), and week 12 (final evaluation). During the screening phase, site investigators contacted all potential participants by phone to assess eligibility for study inclusion.

At the screening visit, eligible participants signed the informed consent form and then underwent a thorough medical examination. An in-person semi-structured interview was performed by site investigators to record data on demographics, medical history, and full clinical course. In addition, the following study procedures were performed: electrocardiography, pregnancy testing, and blood sampling. Patients were instructed to complete a 4-week headache diary in paper format to establish headache characteristics and medication use (available in Supplement 2). At least 80% headache diary compliance was required to enter the open-label treatment phase.

During the open-label treatment phase, patients received 140-mg erenumab monthly by two subcutaneous 1-mL injections at study visits on baseline (day 1), week 4, and week 8. For efficacy and safety assessments, patients were asked to record information daily using a headache diary in paper format. At the follow-up visits (weeks 4, 8, and 12), protocol-specified study procedures were performed, and site investigators assessed efficacy and safety as well as headache diary compliance. At least 80% compliance was required throughout the open-label treatment phase.

### Outcomes

The primary outcome measure was the mean change in number of monthly headache days of moderate to severe intensity from baseline (4-week pretreatment period) to week 9–12. Secondary outcome measures of efficacy included *1)* the mean change in number of monthly headache days of any intensity from baseline to week 9–12, *2)* the proportion of patients achieving at least 50% reduction in the mean number of monthly headache days of any intensity from baseline to week 9–12, *3)* the proportion of patients achieving at least 25% reduction in the mean number of monthly headache days of any intensity from baseline to week 9–12, *4)* the proportion of patients achieving at least 75% reduction in the mean number of monthly headache days of any intensity from baseline to week 9–12, *5)* the mean change in disability score from baseline to week 12, as measured by the Headache Impact Test (HIT-6). Tolerability and safety endpoints were adverse events (i.e. number and type).

### Statistical analysis

Efficacy outcomes measures were calculated based on headache diary entries and analyzed using a complete-case analysis. The latter included patients who received all three doses of erenumab and had at least 80% compliance throughout the open-label treatment phase. The tolerability and safety analyses included all patients who received at least one dose of erenumab. Adverse events were tabulated as frequency counts. R statistical software version 3.6.0 was used to generate all data listing, summaries, and statistical analyses.

### Role of the funding source

The trial was initiated by site investigators who were also responsible for data collection. All authors interpreted the data and contributed to the manuscript preparation, with support from employees of the study funder. Furthermore, all authors made the final decision to submit the manuscript for publication and attest to the accuracy and completeness of the data and reporting of adverse events. The study funder (Novartis Healthcare A/S) did not have the right to veto publication or to control the decision regarding to which journal the paper was submitted.

## Results

### Study participants

**Fig. 1:**
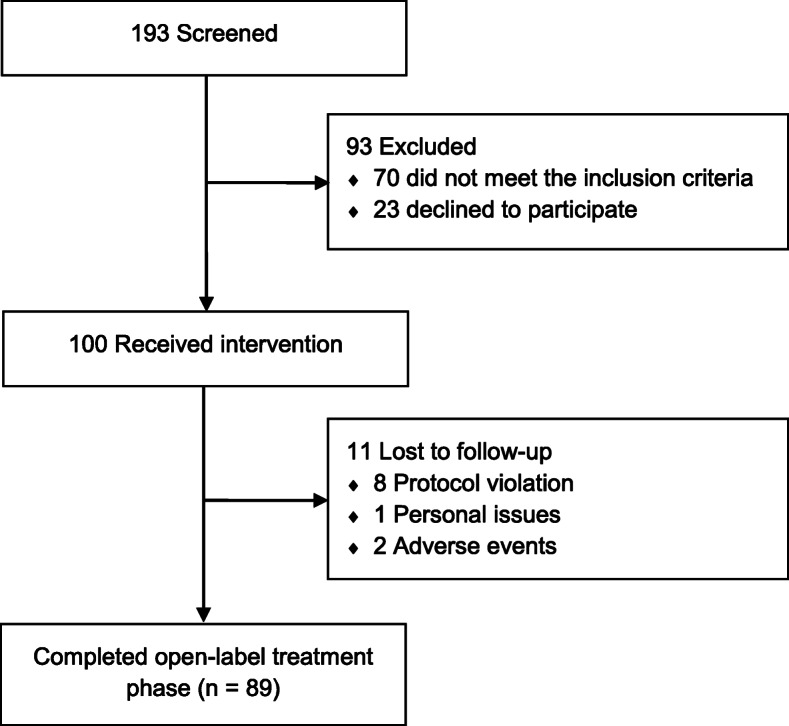
Flow of Participants in an Open-Label Study of Erenumab for Prevention of Persistent Post-Traumatic Headache attributed to Mild Traumatic Brain Injury

A total of 193 patients with persistent PTH were screened for eligibility (Fig. 1), with 23 patients who declined to participate and 70 patients who did not fulfill the eligibility criteria, mostly due to a history of whiplash injury, pre-trauma primary headache disorder, or medication-overuse headache. Thus, 100 patients (75 females and 25 males) were enrolled and received at least one dose of 140-mg erenumab. Table 1 summarizes baseline demographics and clinical characteristics. The mean age (SD) was 35.1 (11.3) years while the mean body mass index (SD) was 25.9 (5.3) kg/m^2^. In terms of employment status, 38% were full-time employed whereas 39% were part-time employed, 21% were unemployed, and lastly, 2% had retired from the workforce. The majority had either a bachelor’s degree or higher education (58%) while 14% had no education besides completion of secondary school or high school. Moreover, 39% had ongoing litigation, whereas 39% as well had ended litigation. Lastly, 19% had a history of pre-trauma psychiatric illness.

**Table 1:** Baseline Participant Characteristics

Characteristics	Persistent PTH (n = 100)
**Age**, mean (SD), y	35.1 (11.3)
**Male/Female**, %	25/75
**Body Mass Index,** mean (SD), kg/m^2^	25.9 (5.3)
**Employment Status**	
Full-time employed, %	38
Part-time employed, %	39
Unemployed, %	21
Retired, %	2
**Education**	
Years of education, mean (SD), y	14.7 (2.9)
No education besides completion of secondary school or high school, %	14
Skilled labor, %	28
Bachelor’s degree, %	31
Higher education, %	27
**Injury Cause**	
Fall, %	33
Motor vehicle collision, %	25
Sports-related injury, %	18
Violence/assault, %	5
Other unintentional injury, %	19
**Disease History**	
Time since mild traumatic brain injury, mean (SD), month	59 (54)
Current acute medication use, No.	89
Current preventive medication use, No.	43
History of preventive medication use, No.	74
No drug failures, %	14
Failure of ≥1 drug, %	86
Failure of ≥2 drugs, %	57
Failure of ≥3 drugs, %	35
Failure of ≥4 drugs, %	19
**Satisfaction with Current Treatment Status**, %	21
**Self-Rated Health**	
Excellent, %	4
Great, %	13
Good, %	40
Rather poor, %	30
Poor, %	13
**Medico-Legal Issues / Litigation**	
Ongoing litigation, %	39
Ended litigation, %	39
Improvement in headache following end of litigation, No. (%)	2 (5.1)
**Headache Phenotypes**	
Chronic migraine-like, %	53
Episodic migraine-like, %	1
Episodic migraine-like combined with chronic TTH-like, %	27
Episodic migraine-like combined with frequent TTH-like, %	6
Chronic TTH-like, %	13
**Aura**, %	11
**Family History of Primary Headache Disorders**, %	31

The most common headache phenotypes were chronic migraine-like headache (53%) followed by combined episodic migraine-like/TTH-like headache (34%), and ‘pure’ chronic TTH-like headache (13%). Current use of acute headache medication was reported by 89%, whereas a history of preventive medication use was reported by 74%. Of the latter, approximately one-fifth had failed at least four preventive medications. Overall, 79 of 100 patients were dissatisfied with their current treatment status.

A total of 89 of the 100 included patients completed the open-label treatment phase and provided data for the complete-case analysis of efficacy outcome measures. Of the 11 patients who did not complete the open-label treatment phase, eight patients were excluded due to protocol violations, i.e. lack of compliance with daily entries in the headache diary (n = 4), logistical issues (n = 3), and unwillingness to maintain stable dosing of a concomitant preventive medication during the treatment phase (n = 1). In addition, another patient withdrew due to personal issues related to a stressful life events, while two patients were excluded due to adverse events (n = 1, worsened headache; n = 1, dizziness).

### Efficacy

**Fig. 2:**
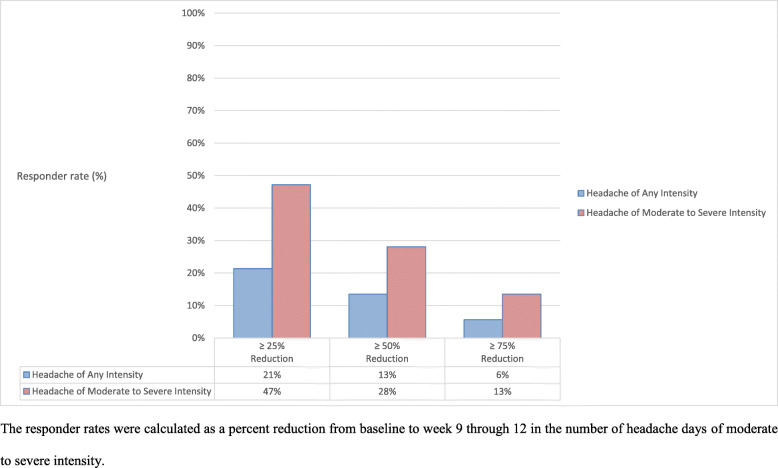
Overview of 25%, 50%, and 75% Responder Rates. The responder rates were calculated as a percent reduction from baseline to week 9 through 12 in the number of headache days of moderate to severe intensity

At baseline, the mean number of headache days of moderate to severe intensity was 15.7 ± 9.6 days per month; by week 9 through 12, the number was reduced by 2.8 ± 6.8 days (Table 2). A post-hoc analysis revealed that a ≥ 50% reduction in the mean number of headache days of moderate to severe intensity was achieved for 28% of the patients (Fig. 2). Furthermore, 47% had achieved ≥25% reduction whereas 12% had achieved ≥75% reduction in mean number of headache days of moderate to severe intensity (Fig. 2). Moreover, patients were categorized for post-hoc subgroup analysis as follows: treatment-naïve patients (n = 24) and patients with at least 2 preventive treatment failures (n = 36). In the former group, erenumab resulted in a reduction of 0.9 ± 9.0 headache days of moderate to severe intensity; by week 9 through 12. The corresponding reduction was 3.0 ± 5.2 headache days of moderate to severe intensity in patients with at least 2 preventive treatment failures.

**Table 2:** Summary of Pre-Treatment Disease Characteristics and Outcome Measures

	Persistent PTH (n = 89)
**Disease Characteristics during 28-day Pre-Treatment Phase**	
Headache days of any severity, mean (SD)	24.6 ± 6.1
Headache days of moderate to severe intensity, mean (SD)	15.7 ± 9.6
Days with use of any acute headache medication, mean (SD)	4.0 ± 4.4
HIT-6 score, mean (SD)	61.6 ± 5.2
**Primary Outcome Measure**	
Mean change in number of monthly headache days of moderate to severe intensity from baseline to week 9–12 (SD)	−2.8 (6.8)
**Secondary Outcome Measures**	
Mean change in number of monthly headache days of any intensity from baseline to week 9–12 (SD)	−1.7 (6.9)
≥ 25% reduction in mean monthly headache days of any intensity, baseline to week 12, %	21
≥ 50% reduction in mean monthly headache days of any intensity, baseline to week 12, %	13
≥ 75% reduction in mean monthly headache days of any intensity, baseline to week 12, %	6
Mean change in HIT-6 score from baseline to week 12 (SD)	−4.6 (7.3)
**Post-Hoc Explorative Outcome Measures**	
≥ 25% reduction in mean monthly headache days of moderate to severe intensity, baseline to week 12, %	47
≥ 50% reduction in mean monthly headache days of moderate to severe intensity, baseline to week 12, %	28
≥ 75% reduction in mean monthly headache days of moderate to severe intensity, baseline to week 12, %	13
Mean change in number of monthly days using acute headache medications, baseline to week 9–12 (SD)	−0.4 (5.2)

The mean number of headache days of any severity was 24.6 ± 6.1 days per month at baseline; by week 9 through 12, the number was reduced by 1.7 ± 6.9 (Table 2). The 50% responder rate for headache days of any intensity was 13% by week 9 through 12, whereas 21% achieved a ≥ 25% reduction and 6% achieved a ≥ 75% reduction (Fig. 2).

At baseline, mean number of days per month with use of acute headache medication was 4.0 ± 4.4 (Table 2). By week 9 through 12, the number was reduced by 0.4 days per month. Mean HIT-6 scores were 61.6 ± 5.2 at baseline and 57.0 ± 8.2, yielding a reduction of 4.6 points (Table 2). Of 89 patients, 44% achieved a ≥ 5-point reduction in HIT-6 score from baseline to week 9 through 12.

### Tolerability and safety

Overall, 100 patients received at least one dose of erenumab and were included in the tolerability and safety analyses (Table 3). Seventy-eight patients reported at least one adverse event, with the most common ones being constipation (*n* = 30) and injection-site reactions (*n* = 15). Of the former, nine patients reported recurrent episodes of constipation. No serious adverse events were reported, although two patients experienced adverse events (dizziness and worsened headache) that led to treatment discontinuation.

**Table 3:** Adverse Events during the 12-Week Open-Label Treatment Phase

Adverse Events	Persistent PTH (n = 100)
**All Events**	
≥ 1 Adverse Event	78
≥ 1 Treatment-Related Adverse Event	38
≥ 1 Serious Adverse Event	0
Any Adverse Event leading to Study Discontinuation	2
**Adverse Events occurring in ≥ 2% of Patients**^**a**^	
Injection-Site Reactions	
Pain	7
Erythema	5
Hemorrhage	3
Acid Reflux	1
Constipation	30
Diarrhea	2
Dizziness	9
Dry Mouth	4
Fatigue	5
Hot Flashes	3
Influenza	2
Irregular Menstruation	3
Low Back Pain	2
Nausea	7
Palpitations	2
Upper Abdominal Pain	5
Worsened Headache	8

Data are reported as number of patients. If a patient had the same adverse event more than once, it was counted only once

## Discussion

Treatment with 140-mg erenumab yielded a reduction of 2.8 headache days of moderate to severe intensity by week 9 through 12, while the rate of a 50% or greater reduction was 28%. In those with at least 2 preventive treatment failures, the corresponding reduction was 3.0 headache days of moderate to severe intensity. Interestingly, one randomized clinical trial (RCT) found that erenumab yielded a reduction of 1.8 migraine days by week 9 through 12 in patients with high-frequency episodic migraine who had failed at least 2 preventive treatments [16]. Of note, the same study found that the corresponding reduction was only 0.2 migraine days in the placebo group [16]. Although our results cannot be directly compared with those of erenumab trials in migraine, it seems reasonable to draw some comparisons considering recently published data [6]. In a study of 91 patients with persistent PTH who had a migraine-like phenotype, the mean monthly number of migraine-like days was 14.5 [6]. Similarly, the mean monthly number of headache days of moderate to severe intensity was 15.7 at baseline in the present study population. Thus, it is likely that headache days of moderate to severe intensity largely reflect migraine-like headache days in patients with persistent PTH.

Overall, erenumab was well-tolerated, with most patients (89%) receiving all three planned doses of erenumab. No safety concerns were found and only two patients discontinued the treatment regimen due to adverse events. The most frequently reported adverse events were constipation and injection-site reactions. Of note, nine of the 100 included patients reported recurrent episodes of constipation. However, this finding is of limited use as we did not record any data on the occurrence of occasional constipation prior to treatment with erenumab. In general, long-term data is needed to confirm the tolerability and safety of erenumab in individuals with persistent PTH.

Efficacy assessments are complicated by lack of placebo comparison. Thus, our findings should be interpreted with caution, although they provide context to observations made by clinicians who currently use erenumab as an off-label preventive treatment for individuals with persistent PTH. Moreover, it should be mentioned that 74 of the 100 included patients reported a history of preventive medication use. Of these, 86% had failed at least one preventive medication. It could be speculated that the placebo response is lower in those with history of preventive medication failure. Indeed, ample data from migraine trials with erenumab have consistently shown a lower placebo response in patients who reported previous failure of preventive medications [16–18]. As a migraine-like headache phenotype was present in 87 of the 100 included patients, it should be reasonable to assume that a similar placebo response is found in individuals with persistent PTH.

An important implication of the present study should be a future emphasis on use of standardized outcome measures and a requirement of subjects to fulfill the ICHD criteria for PTH [15]. This might facilitate comparative assessments and reduce heterogeneity between study populations. Similar conclusions were also made in a recent systematic review of preventive treatments for PTH [5]. Indeed, the authors were not able to infer efficacy of any preventive treatment due to an absence of placebo-controlled RCTs and a lack of high-quality open-label studies. In addition, efficacy outcomes had not been prospectively defined and varied between studies [19–21]. We would recommend that future intervention studies include prospectively defined outcomes measures that are documented using a headache diary. Furthermore, delineation of effective and well-tolerated preventive treatments requires intervention studies that apply a placebo-controlled RCT design. In this context, erenumab and other anti-CGRP monoclonal antibodies hold great promise as a growing body of evidence suggests CGRP involvement in the pathogenesis of PTH [12, 13]. In particular, it would be very intriguing to assess whether treatment in the early phase following TBI could prevent development of persistent PTH at 3 months post-trauma.

### Limitations

This study has several limitations. First, the study included an open-label design, without a placebo arm. As a result, it is difficult to interpret the efficacy and possible relatedness of an adverse event. Second, outcome measures were evaluated at a short-term follow-up of 3 months after the first dose of erenumab. Consequently, long-term observational studies are needed to adequately ascertain the efficacy and safety of erenumab. Third, assessment of previous treatment failures was done retrospectively using a semi-structured interview which may introduce recall bias. Fourth, efficacy outcome measures were evaluated using a headache diary in paper format, which does not allow monitoring of daily entries.

## Conclusions

The present study suggests that erenumab might be a useful preventive treatment for persistent PTH. It appears that erenumab primarily reduces headache days of moderate to severe intensity, which often mimic the features of a migraine-like headache. As discontinuations due to adverse events were low, further research is much needed to assess the effectiveness of erenumab against placebo as well as other preventive medications.

## Data Availability

Qualified researchers can request access to patient-level data and related study documents, including the study protocol. Patient-level data will be de-identified and study documents will be redacted to protect the privacy of trial participants.

## Abbreviations

PTH: Post-traumatic headache
TBI: Traumatic brain injury
CGRP: Calcitonin gene-related peptide
ICHD-3: International Classification of Headache Disorders 3rd edition
TTH: Tension-type headache
HIT-6: Headache Impact Test
RCT: Randomized clinical trial
